# Prevalence of DSM-V mental disorders in a cohort of young adults in Ireland

**DOI:** 10.1192/bjo.2021.720

**Published:** 2021-06-18

**Authors:** Josen McGrane, Eleanor Carey, Emmet Power, Niamh Dooley, Sean Madden, Helen Coughlan, Donal Campbell, Mary Clarke, Mary Cannon

**Affiliations:** 1 Royal College of Surgeons in Ireland, St Vincent's University Hospital; 2 Royal College of Surgeons in Ireland, St Vincent's University Hospital, Trinity College Dublin; 3 Trinity College Dublin, Royal College of Surgeons in Ireland; 4 Independent Scholor; 5 Royal College of Surgeons in Ireland; 6 Beaumont Hospital; 7 Royal College of Surgeons in Ireland, Beaumont Hospital

## Abstract

**Aims:**

To estimate the prevalence of DSM-V mental disorders in a population of Irish emerging adults

**Background:**

Mental disorders are the leading cause of years lived with disability in youth worldwide. Few studies use gold standard of face to face semi-structured standardized interview tools, and this is a limitation in the estimates of prevalence rates of mental disorder in the extant literature.

**Method:**

Briefly, we recruited a representative sample of 212 adolescents and followed them up over ten years. In this wave of the adolescent brain development study, 103 of the initial 212 participants took part, 50 males and 53 females, with a mean age of 20.87 years (SD = 1.3). Psychopathology was assessed in all participants by trained research psychologists and mental health professionals using the Structured Clinical Interview for DSM-V (SCID).

**Result:**

52.4% of participants had one lifetime mental disorder, the prevalence rates were highest for Major Depressive Episode (25.3%), Social Anxiety (12.6%) and Generalized Anxiety (8.7%). 50.5% had a history of a mental disorder. 27.2% had 1 lifetime diagnosis, 15.5% had 2 and 7.8% had >2.

**Conclusion:**

Rates of mental disorder rapidly increase during emerging adulthood. In a similar Irish study, 55% of young adults met the criteria for lifetime mental disorder. Whilst the rates of mental disorder are high in young people, previous longitudinal research has suggested that many common mental disorders remit by the late twenties. We suggest a need for further research investigating the comparative later functional and economic outcomes of these young people. Research to date is supportive of a need to expand capacity of youth friendly services for prevention and treatment.

Ethical Approval

Ethical approval for the study protocols, including interviews and assessments, along with informed consent documents, was granted by the Beaumont Hospital Medical Ethics Committee in 2016.

Acknowledgements:

1. European Research Council Consolidator Award and Health Research Board Ireland Award to Mary Cannon

2. Health Professionals Fellowship from the Health Research Board Ireland to Helen Coughlan.

